# Ensemble Model for Diagnostic Classification of Alzheimer’s Disease Based on Brain Anatomical Magnetic Resonance Imaging

**DOI:** 10.3390/diagnostics12123193

**Published:** 2022-12-16

**Authors:** Yusera Farooq Khan, Baijnath Kaushik, Chiranji Lal Chowdhary, Gautam Srivastava

**Affiliations:** 1 School of CSE, Shri Mata Vaishno Devi University, Katra 182320, India; 2 School of Information Technology and Engineering, Vellore Institute of Technology, Vellore 632014, India; 3 Department of Mathematics and Computer Science, Brandon University, Brandon, MB R7A 6A9, Canada; 4 Research Centre for Interneural Computing, China Medical University, Taichung 40402, Taiwan; 5 Department of Computer Science and Math, Lebanese American University, Beirut 1102, Lebanon

**Keywords:** neuroimaging Alzheimer’s disease, K-NN, naïve Bayesian, ensemble learning, gradient boost, multi-layer neural network

## Abstract

Alzheimer’s is one of the fast-growing diseases among people worldwide leading to brain atrophy. Neuroimaging reveals extensive information about the brain’s anatomy and enables the identification of diagnostic features. Artificial intelligence (AI) in neuroimaging has the potential to significantly enhance the treatment process for Alzheimer’s disease (AD). The objective of this study is two-fold: (1) to compare existing Machine Learning (ML) algorithms for the classification of AD. (2) To propose an effective ensemble-based model for the same and to perform its comparative analysis. In this study, data from the Alzheimer’s Diseases Neuroimaging Initiative (ADNI), an online repository, is utilized for experimentation consisting of 2125 neuroimages of Alzheimer’s disease (*n* = 975), mild cognitive impairment (*n* = 538) and cognitive normal (*n* = 612). For classification, the framework incorporates a Decision Tree (DT), Random Forest (RF), Naïve Bayes (NB), and K-Nearest Neighbor (K-NN) followed by some variations of Support Vector Machine (SVM), such as SVM (RBF kernel), SVM (Polynomial Kernel), and SVM (Sigmoid kernel), as well as Gradient Boost (GB), Extreme Gradient Boosting (XGB) and Multi-layer Perceptron Neural Network (MLP-NN). Afterwards, an Ensemble Based Generic Kernel is presented where Master-Slave architecture is combined to attain better performance. The proposed model is an ensemble of Extreme Gradient Boosting, Decision Tree and SVM_Polynomial kernel (XGB + DT + SVM). At last, the proposed method is evaluated using cross-validation using statistical techniques along with other ML models. The presented ensemble model (XGB + DT + SVM) outperformed existing state-of-the-art algorithms with an accuracy of 89.77%. The efficiency of all the models was optimized using Grid-based tuning, and the results obtained after such process showed significant improvement. XGB + DT + SVM with optimized parameters outperformed all other models with an efficiency of 95.75%. The implication of the proposed ensemble-based learning approach clearly shows the best results compared to other ML models. This experimental comparative analysis improved understanding of the above-defined methods and enhanced their scope and significance in the early detection of Alzheimer’s disease.

## 1. Introduction

Alzheimer’s disease (AD) can be treated with the use of healthcare informatics. The Alzheimer’s enigma has troubled both researchers and clinicians. A German doctor Alois Alzheimer over a century ago spotted anomalies in brain sections of a patient with dementia. Researchers tried to study these strange anomalies known as plaques and tangles in the brain. The brain with dementia is characterized by wiped memories and intellect getting destroyed, thereby resulting in memory failure [1]. Dementia that attacks memory, self-awareness and rational intellect is accompanied by plaques and tangles in the brain as a part of aging. As Alzheimer’s disease progresses, brain tissues shrink and die, which in turn causes the gradual loss of cognitive ability, difficulty in thinking, decision-making, and confusion, leading to ultimate brain atrophy [2]. Early signs are the building-up of plaques around the neurons. These plaques are made up of proteins which stop their actual functioning as soon as they undergo mutation [3].

The World Health Organization (WHO) has observed that by 2030, the worldwide economic influence of Alzheimer’s disease (AD) will exceed USD 2 trillion per year [4]. According to the Alzheimer’s Association (https://www.alz.org/, accessed on 14 November 2022), the disease has become the 6th foremost reason for death in the United States. More than 16 million individuals are given unpaid care and, predictably, 18.5 billion hours of care costs nearly USD 234 billion. As per the survey, one in three senior citizens died of AD or another dementia [5]. The death rate due to AD is more than the deaths caused by breast cancer and prostate cancer. In 2021, it was predicted to cost USD 355 billion, and by 2050, it might increase to USD 1.1 trillion.

**Figure 1:**
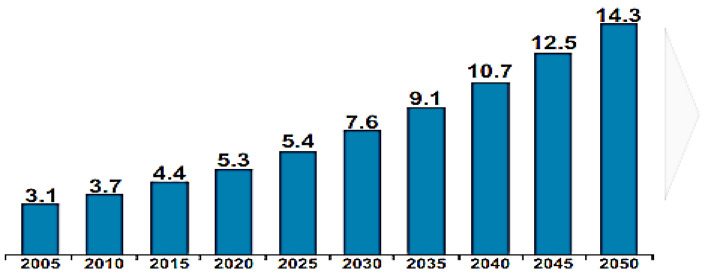
Individuals affected with dementia (in millions) [Source: http://ardsi.org (accessed on 5 May 2022)].

Alzheimer’s & Related Disorders Society of India (ARDSI) [6] predicts that the number of people living with dementia would rise from 7.6 million in 2030 to 14.3 million by 2050, putting an enormous burden on families. Figure 1 represents the estimated Indian population with dementia from 2005 to the near future 2050 taken from ARDSI [6]. Certainly, it is a necessity to detect and predict initial changes in AD so that cost-effective treatments and therapies can be applied to address AD and other related disorders. Despite all advancements in the research to improve our understanding of neurodegeneration, no medication exists [7]. Therefore, it is very important to detect and diagnose this devastating condition as early as possible. AD is a progressive neurodegenerative disorder marked by the build-up of insoluble mutant proteins known as plaques and tangles [8]. Plaques are mysterious peptides called Amyloid-β (Aβ) [9]. These peptides are formed when two enzymes sequentially breakdown a protein called Amyloid precursor protein: β and γ secretase [10]. Additional fragments are formed as a result of this cleavage, which may contribute to the disease’s pathogenesis. Due to its tendency to fold incorrectly, Aβ tends to clump together and form solubilizing polymers [11]. Few of them convert into intricate fibrils known as plaques which accumulate in the brain. Because of plaque deposits, the synapse starts to fail, and irregular configurations of activity emerge. Ultimately, the brain becomes unable to process and store information accurately. An additional significant feature of AD is neurodegeneration, neuronal loss [12], and destruction, which initiates other proteins called Tau [13], which also seem to be a constituent of massive jumbles called tangles. In a brain cell, particles are passed via an axon on a chain of tracks called microtubules. These tubular structures are stabilized by Tau [14]. Although Tau develops in AD, the microtubules that hold it in place are reformed, enabling it to break off and migrate from the axon to the cell body. Like amyloid-β, they also stick to deposition called neurofibrillary tangles. Eventually, these processes kill neurons which is a gradual and irreversible process [15]. Neurodegeneration leads to brain atrophy differentiating the normal brain from the diseased brain.

The most important and familiar tool for understanding and studying neurodegeneration and neurodegenerative diseases is neuroimaging [16] which includes multiple imaging techniques such as computed tomography (CT), dopamine transporter scan (DatScan) magnetic resonance imaging (MRI), positron emission tomography (PET), and Single-photon emission computed tomography (SPECT) [17]. Neuroimaging gives a comprehensive understanding of the potential mechanisms underlying these neurodegenerative diseases such as AD, Parkinson’s disease (PD) [18], Attention-Deficit/Hyperactivity Disorder (ADHD) [19], and Autism Spectrum Disorder (ASD) [20] so that better clinical trials are developed and deployed to improve diagnosis and effective healthcare. These large-scale neuroimaging databases may be used to train and evaluate diagnostics using Artificial intelligence and Machine learning methods for neurodegenerative diseases (e.g., Alzheimer’s disease). Predicting Alzheimer’s disease with machine learning has been the subject of several research studies.

However, scrutinizing previous studies, some gaps are identified related to the diagnosis of AD which encourages us to work in the direction to overcome those drawbacks. Taking this into consideration, this paper presents an effective ensemble-based technique and provides an in-depth comparison of Machine learning methods to predict AD, as well as future research possibilities. Ensemble learning is the process of transforming numerous weak models into a single robust model. This can be achieved using a variety of ensemble strategies, including bagging, boosting, and stacking. Ensemble learning integrates the information to acquire new information and enhances the performance accuracy of predictions done adaptively through voting schemes. The fundamental goal of voting is to increase generalization by balancing individual model errors, particularly when the models perform well on a predictive modelling task. Moreover, in the Ensemble approach, if it is found that the same model performs well when using different hyperparameters, then combining multiple fits of the same machine-learning algorithm is beneficial. When compared to a single model, an ensemble model always provides more accurate results and has greater applicability.

This paper provides a comprehensive evaluation of learning approaches for Alzheimer’s prediction, as well as potential future developments.

The main contributions of this article can be understood through the points mentioned as under:

1. This study evaluated the performance of various machine learning techniques, i.e., 11 models which lack in the state-of-the-art literature.
2. To enhance the diagnostic performance of AD, this article presents an ensemble technique formed by combining SVM kernel master demonstration, along with extreme gradient boosting transitioning sequential model to concur the hidden difficult extractions in the dataset. The proposed architecture (XGB + DT + SVM) uses a Decision Tree to reduce invariability in model demonstration and performance. Overall, the proposed model not only decreases inheriting variance but enhances tough sample prediction using boosting.
3. This article performed a comparative analysis of algorithms before and after hyper parameter tuning accomplished through Grid search and the outcomes are validated using k-fold cross-validation.
4. To ensure the generalization of the considered models, this article evaluated the efficiency of the same using statistical test, i.e., Friedman’s rank test and *t*-test.
5. Various performance metrics such as receiver operating characteristics (ROC) curve, sensitivity, specificity and log loss are used to compare the proposed model with other ML models.
6. This article also performed a comparison of the proposed model with existing state-of-the-art studies to prove its efficiency in detecting AD.
7. The article also focuses on the future scope that should be addressed for reliable AD diagnosis.

The remaining part of the paper is organized as follows: Section 2 discusses related work. Section 3 discusses the existing approaches and dataset. Section 4 presents a description of the experimental analysis. Section 5 discusses the comparative performance evaluation of each method of the framework and Section 6 concludes the paper following the future work in Section 7.

## 2. Related Work

As with other neurodegenerative illnesses, Alzheimer’s disease causes persistent and irreversible cognitive deterioration, culminating in brain shrinkage. It is an expensive medical condition that places a substantial financial strain on families and care givers. As neurodegeneration is an irreversible process, it cannot be treated. For this reason, researchers are focusing on the early identification and prediction of neurodegeneration to give optimal treatment to those suffering from neurodegenerative disorders. Substantial research has been performed and continues to be conducted in this field to enhance the early identification and prediction of a better diagnostic assistance system.

Roman Casanova et al. [21] used a large-scale regularization approach based on logistic regression to classify MRI scans to determine a person’s level of thinking and reasoning. The authors used a regularization approach on a dataset of cognitively Normal Controls (NC) and disease patients (AD). Their results showed 85.7% sensitivity, 82.9% specificity, and 90% accuracy, respectively. In a study by Costafreda et al. [22], MRI data were used to identify people with mild mental impairment who were at risk of developing Alzheimer’s disease. The authors used SVM to classify mild cognitive impairment as Alzheimer’s disease, with 80% and 77% sensitivity, respectively. Battineni et al. [23] tested four machine learning (ML) models, including NB, ANN, K-NN, and SVM, and the receiver operating characteristic (ROC) curve metric was used to verify the model performance. Each model was evaluated in three separate experiments. Firstly, the models were trained on manually selected features, and ANN produced the greatest ROC accuracy (0.812). In the second experiment, automated feature selection was performed via wrapping approaches, with the NB achieving the greatest ROC of 0.942 and finally, the ensemble model showed an ROC of 0.991.

Padilla et al. [24] examined neuroimaging data from MRI, PET, and SPECT scans of healthy and sick subjects. As a result, they used the Fisher discriminant ratio (FDR) and non-negative matrix factorization (NMF) to identify and categorize the most significant features. Their method yielded a 90% accuracy rate. Xin Liu et al. [25] investigated structural-MRI by using locally linear embedding (LLE) to transform multivariate MRI data into linear space, focusing on regional brain volume and cortical thickness. In this study, LLE was found to be useful in identifying Alzheimer’s disease. Gray et al. [26] performed a multi-modal classification of MRI data, focusing on MRI volumes and voxel-based PET signal intensities. Heung-II Suk et al. [27] proposed a deep learning feature-based strategy. The authors concentrated on grey matter tissue volumes, which are low-level MRI characteristics. They also used PET signal intensities in their method. The dataset was obtained from ADNI, and the experimental results had a 95.9% accuracy rate. Juergen Dukart et al. [28] proposed the use of SVM on information derived from the combination of MRI and FDG PET for detecting frontotemporal lobar degeneration. Using the ADNI dataset to train the classifier, they achieved 91% classification accuracy. Combining MRI and FED-PET findings may better determine Alzheimer’s disease (AD).

Liu et al. [29] introduced a novel hierarchical technique combining multi-level classifiers resulting in ensemble classification. They continued to add features from various parts of their brains until they were all in one place. They used imaging parameters such as spatially normalized grey matter (GM) from each MRI scan to achieve an accuracy of 92.0%. Authors presented MK-Boost to classify ADNI data using a Whole-Brain Hierarchical Network (WBHN) based on an Automated Anatomical Labelling atlas (ALL). With an AUC of 0.907, its AD/MCI classification model was 89.53% accurate. Tingting et al. [30] proposed a multimodality-based classification of Alzheimer’s disease and mild cognitive impairment (MCI). They used a linear regression model with group sparsity regularization on weights from the ADNI dataset to improve classification performance and identify syndrome-associated biomarkers useful for illness analysis. Zu et al. [31] classified Alzheimer’s disease and mild cognitive impairment using a new multimodal technique. As a result, label-aligned multiple-task feature extraction and multimodal categorization have been added. They combined selected multimodality characteristics using a multi-kernel SVM. They achieved 95.05% classification accuracy using the ADNI MRI and FDG-PET datasets.

According to Sorensen et al. [32], variations in volumetric alterations contribute to early cognitive decline. T1-weighted MRI images were analyzed from the ADNI database. Liu et al. [33] used a blend of various kernels to link edge characteristics with node architecture while analyzing MRI data from the Alzheimer’s disease Neuroimaging Initiative (ADNI). Their investigation discovered that 3D texture was more effective for categorization findings, with an accuracy of 91%. Wang et al. [34] used two data sources, from the OASIS and the Washington University School of Medicine. They formed an ethical committee and signed consent from each subject before entering the study. The study included four native hospitals.

Table 1 depicts a summary of existing work using MR imaging in Alzheimer’s disease categorization using multiple techniques

The after section comprises the methodology that we used for the classification of AD and MCI using different categories of algorithms.

## 3. Materials and Methods

This section comprises the methodology for comparative analysis of classification algorithms for predicting individuals with Alzheimer’s disease and mild cognitive impairment. The given procedure consists of different phases: (i) collection of MRI data (ii) classification, and (iii) comparison of performance among these algorithms.

In Section 3.1, the framework of this study is addressed. In Section 3.2, a description of the ADNI dataset, data pre-processing and feature extraction is given. In Section 3.3, we discussed the different learning techniques to identify individuals with AD and MCI.

### 3.1. Framework

**Figure 2:**
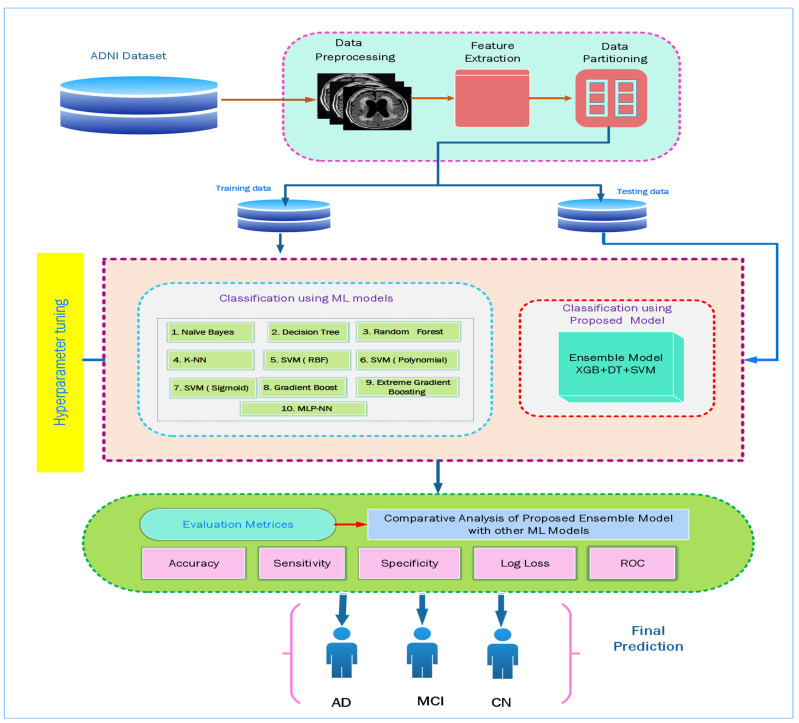
Framework for Alzheimer’s disease classification and prediction using various algorithms.

Figure 2 presents the framework of the study. With the advent of ML throughout the last couple of years, a variety of methods have been introduced to enhance AD classification using neuroimaging as a biomarker. In this study, we used the axial MR Neuroimaging modality, and the dataset is divided into Train-set (70%) and Test-set (30%). Classification algorithms are trained for three classes of data by providing a training-set as input.

### 3.2. Dataset Description

Neuroimaging data were obtained from the ADNI website (http://adni.loni.usc.edu/ (accessed on 7 September 2022)). The ADNI initiative was launched by Dr. Michael W. Weiner as its primary investigator, at understanding the evolution from usual ageing to insignificant memory problems in Alzheimer’s disease. Along with the neuroimaging initiative, they also process the genetic and clinical information to analyze and measure various genes for determining the extent of genetic risk participating in the increase in the Alzheimer’s disease enigma. In this study, we have taken 2127 MRI (T1 and T2 weighted) data from ADNI. The downloaded dataset is categorized into three classes: 612 samples for the AD class, 538 samples for MCI, and 975 samples for the CN class as shown in Table 2.

#### 3.2.1. Pre-Processing Steps

**Figure 3:**
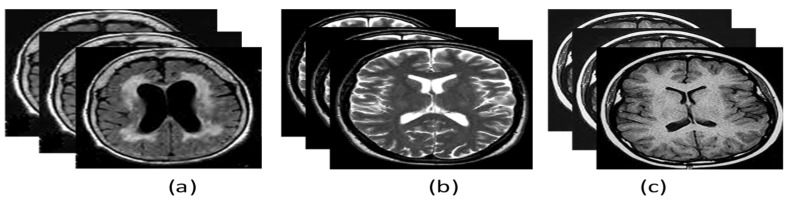
Brain axial MRI (T1 and T2 weighted) data for (**a**) Alzheimer’s Disease, (**b**) Mild Cognitive Impairment and (**c**) Control Normal.

The data taken from the ADNI database require some basic tasks before it is presented to the algorithms:

(a) Data filtering is performed based on the acquisition plane and ADNI phase. We selected the axial acquisition plan of the MR (T1 and T2 weighted) images and ADNI phase 3 for three classes: AD, CN, and MCI as shown in Figure 3.
(b) The images are reshaped to 512 × 512 to provide the best quality pixel.
(c) The labels about the classes of the images are extracted and put into the vector storage. The classes undergo label encoding, i.e., 0 for AD, 1 for MCI and 2 for CN.
(d) The loaded images are standardized and normalized using a standard scaler and min-max scaler as the normalization functions.
(e) Once the preprocessing of the data is completed, the data are split into training and testing to perform validation on unseen data.

**Figure 4:**
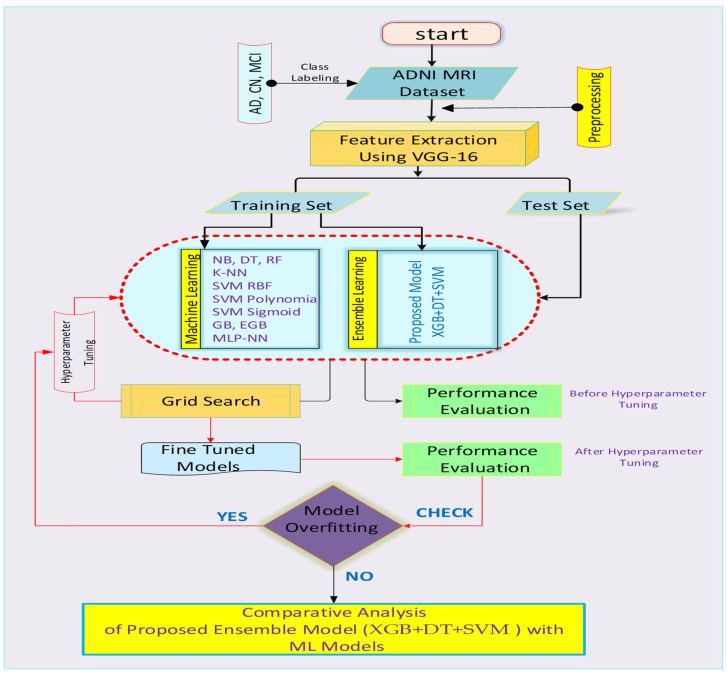
The flow chart depicts the flow of the study.

The MRI axial scans are fed into the model as input, which is then converted into NumPy arrays. A NumPy array is an n-dimensional array that is represented as a grid of values with non-negative index tuples. The rank of the array gives the number of dimensions, and the shape of the tuple gives the array size. Figure 4 given below presents the workflow of this study as discussed below.

1. Data: Brain axial T1 and T2 weighted MRI data taken from the ADNI database is labelled into three classes such that AD CN, and MCI along with the preprocessing and data loading pipelines.
2. Data training: using VGG-16 [43] as a feature extractor primarily using the illustrations provided by the Imagenet on the ADNI dataset. After Feature Extraction using VGG-16, the extracted blob is then processed and sent to conventional machine learning models and proposed ensemble architecture.
3. Parameter optimization: post-training hyperparameter tuning is achieved using GridSearch.
4. Data Evaluation: after rigorous training, the models are tested and compared for overfitting. If overfitting is found, then retraining is done with reduced hyper-planes and data sampling. Else, a comparative analysis of the result is evaluated.

#### 3.2.2. Feature Extraction

Feature Extraction is an integral aspect of image-based machine learning as it allows for the inculcation of the feature-wise similarity graph or correlation table available to the deep neural architectures such as VGG-16 that is utilized in this paper. In this study, a freezing bottleneck of VGG-16 is implemented, with the initial convolutional layer extraction being transformed into dense arrays of 1000 vectors for use in the machine learning proposition. In comparison to local filtering or Gaussian noise extraction methods, this method accelerates convergence and improves accuracy by 31.27% in aggregate. VGG-16 uses the internal feature extractor using images of size 100 × 100 × 1 which is self-convoluted 100 × 100 × 3 using 3 filtered convolutions with padding maintained at the same value. Further, the kernel association blob that is predicted or processed by the VGG-16 architecture is 2 × 2 × 512, when linearized using flatten and dense layers it is converted to 4096 vectors. This blob is fed into traditional machine learning models and the suggested ensemble architecture, from which AD, CN, and MCI predictions are derived. The proposed VGG-16 model uses label-encoded vectors for dependent variables and cross-channel normalization. In VGG-16, modelling, the batch size is 32, and the loss function is Cross-entropy loss. The feature stimulator extracts image information using 1 × 1, 3 × 3, and 5 × 5 kernels, as well as the gallort weight initializer and pre-trained Imagenet data.

### 3.3. Classification Models

In this subsection, classical machine learning models are discussed in brief. The mathematical background of various classical learning models discussed includes Decision Tree (DT), Random Forest (FR), Naïve Bayes (NB), K-Nearest Neighbor (K-NN), SVM (RBF kernel), SVM (Polynomial Kernel), SVM (Sigmoid kernel), Gradient Boost (GB) and Extreme Gradient Boosting (XGB).

#### 3.3.1. Naive Bayes (NB)

NB is a machine learning algorithm whose work is based on the standards of the Bayes theorem named after Thomas Bayes which is based on conditional probability [44,45]. It is used as a classifier in face recognition, weather prediction, news classification, and medical diagnosis. The posterior probability in this algorithm is calculated using Equation (1).

(1) $$P\;(C|X)=\frac{(P\left(X|C\right)P\left(C\right)}{P\left(X\right)}$$

Equation (1) above is used to depict posterior probability P (C|X) where *C* is a class variable and *X* is a dependent feature vector. The posterior probability is denoted by P (C|X), likelihood is denoted by (P(X|C) class prior probability by P(C) and predictor prior probability by P(X).

#### 3.3.2. Decision Tree (DT)

DT classifier has the inherent capability of eliminating useless elements from the input data, thereby increasing the performance of the algorithm [46]. The classification technique is based on non-parametric supervised learning, which predicts the class of given data by learning features from data in the form of a tree structure. This classification technique is used for predicting the type of class to which our data belong. Thus, it supports diagnosis decisions by dividing the dataset into smaller sets to specify a sequence of decisions and consequences. Numerous metrics can be used to determine the optimal method for splitting the records. These metrics are described in terms of pre-splitting and post-splitting class distributions of the documents. p(i|t) represents the percentage of records from class 1 located at node t. The degree of impurity in the child nodes influences several measures used to determine the ideal split. The degree of impurity is proportional to how distorted the distribution of classes is. Impurity measurements are given in Equations (2)–(4).

(2) $$\mathrm{Entropy}\;(t)=-\sum_{i=0}^{c-1}\;p\left(i|t\right){log}_{2}\left(i|t\right)$$

(3) $$\mathrm{Gini}\;(t)=1-\sum_{i=0}^{c-1}{\left[p\left(i|t\right)\right]}^{2}$$

(4) $$\mathrm{Classification}\;\mathrm{error}\;(t)=1-max_{i}\left[p\left(i|t\right)\right]$$

The input features for the DT models can be selected using Entropy (t) as stated in Equation (2), Gini Index (Gini (t)) using Equation (3) and the Classification error (t) calculated using Equation (4). In the above Equations, c is the number of classes.

#### 3.3.3. Random Forest (RF)

Random Forest (RF) [47] is a classifier that works as a group of decorrelated decision trees to perform classification. It was created using multiple decision trees. RF is best used for medical diagnosis, remote sensing, and object detection in images. RF finalizes the decision by estimating average votes from different trees. The matrices containing sample features of the training dataset are given as input to the algorithm. For each sample, a DT is created to make a classification decision and the significance of every feature in each tree is computed. The prediction in RF is taken as an average of the trees and the formula in RF is given in Equation (5):

(5) $$F\left(x\right)=\frac{1}{j}\;\overset{j}{\underset{j=1}{\sum }}c_{jfull}+\overset{k}{\underset{k=1}{\sum }}(\;\frac{1}{j}\sum_{j=1}^{j}contribution_{j}\;\left(x,k\right))$$

where F(x) is the prediction function, *j* is the number of trees in the RF model and k is the total number of features.

#### 3.3.4. K-Nearest Neighbor (K-NN)

The K-NN algorithm [48] works on the principle of the similarity measure. The number of available cases or nearest neighbours that contribute to voting is represented by K and the model organizes new data depending on similarity with the available cases. The algorithm is mostly used in search applications where the task is to search for similar data objects. The probabilistic interpretation of K-NN is discussed using Equations (6)–(8).

(6) $$P\left(x\right)=\frac{k}{NV}$$

(7) $$P\left(x|y\right)=\frac{k_{1}}{N_{1}V}$$

In Equations (6) and (7), the nearest neighbour from class 1 is denoted by *k*_1_ and *N*_1_ signifies the total sum of instances in class 1.

(8) $$p\left(x\right)=\frac{k}{N\;C_{d}\;R_{K}^{D}\left(x\right)}$$

In Equation (8), $R_{K\;}^{D}$(*x*) is the distance between the estimation point and the *k*th closest neighbour and $C_{d}$ represents the volume of the sphere in the *D* dimension.

#### 3.3.5. Support Vector Machine (RBF Kernel)

Support vector machine methods analyze data and sort them into categories based on supervised learning. SVM (RBF kernel) follows the use of a labelled training dataset as input to learn the classifier and produce input-output mapping functions [49]. Support vector machines are versatile because diverse kernel functions can be listed for the judgment functions. It is possible to define common kernels as well as custom kernels. RBF stands for the Radial Basis function kernel that works in infinite dimensions. Radial kernel SVM was used to deal with overlapping data. The radial kernel is mathematically represented in Equation (9).

(9) $$k=e^{-\gamma {\left(a-b\right)}^{2}}=\exp(\frac{-{\left|\left|x-y\right|\right|}^{2}}{2\sigma^{2}})$$

The kernel *k* determines the influence of each observation in the training dataset as stated in Equation (9). *γ* (gamma) is determined by cross-validation scales, perceived as an inverse of the radius.

#### 3.3.6. Support Vector Machine (Polynomial Kernel)

SVM (Polynomial Kernel) calculates a high-dimensional relationship between observations [50,51]. A polynomial kernel is mathematically represented in Equations (10) and (11).

(10) $$k={\left(a\times b+r\right)}^{d}$$

For *d* = *n*,

(11) $$k={\left(\sum_{i=1}^{n\;}x_{i\;}y_{i\;}+r\right)}^{n}$$

In the above Equations (10) and (11), *a* and *b* define different observations in the dataset, *r* defines the polynomial coefficient, and *d* sets the degree of the polynomial. The polynomial method sacrifices the linearity and uses higher-degree polynomial equations for higher-dimensional data.

#### 3.3.7. Support Vector Machine (Sigmoid Kernel)

SVM (Sigmoid Kernel) [52] is known by the name hyperbolic tangent kernel and also as a multilayer perceptron kernel. The sigmoid function is used for activation, mathematically represented as:

(12) $$k(x_{i},\;x_{j}=\tanh\left(\alpha x_{i}{}^{t}x_{j}+c\right)$$

In Equation (12), *c* is the intercepting constant and α is the slope. *x_i_* is the ith instance and *x_j_* is the *j*th feature of the *i*th instance. The Support vector sigmoid kernel is interesting because of its origin in neuronal network theory and has been found to perform well.

#### 3.3.8. Gradient Boost (GB)

GB [53] is also founded on the theory of sequential ensemble or collection of predictors where the basic learners are created progressively in such a way that the existing base learner should be more competent than the previous one. Predictions are combined by weighted average or vote to provide an overall prediction. The goal of boosting algorithms is to minimize the empirical loss functional as given in Equation (13):

(13) $$L\left(f\right)=\frac{1}{m}\overset{m}{\underset{i=1}{\sum }}\Phi \;(y_{i,\;\;}\;f\left(x_{i}\right))$$

In Equation (15), L(f) is an empirical loss function and is viewed as Functional Gradient Descent rather than a vector-valued function to minimize the loss *L*. *x_i_* is a training sample of *m* size.

#### 3.3.9. Extreme Gradient Boosting (XGB)

The XGB classifier [54] is commonly used for complex and more convoluted data-driven real-world problems. Boosting comes under the category of ensemble learning techniques where weak learners are combined to become strong learners so that the accuracy of the model is increased. Thus, boosting is an efficient method for increasing the performance of classification. It is possible to execute an ensemble in one of two ways: using a sequential ensemble model, also known as boosting, where the weak learners are created sequentially during the training phase or using a parallel ensemble model, also known as bagging, where the weak learners are produced in parallel during the training phase. Mathematically, boosting can be represented with the help of Equation (14).

(14) $$\;f_{1}\left(x\right)<-f_{0}\left(x\right)+h_{1}\left(x\right)$$

where *f*_0_ is considered the initial model to predict the target variable. *h*_1_ is used to fit the residual from the preceding steps.

The performance can be further improved by building a new model after the residuals of *f*_1_ as shown in Equation (15).

(15) $$f_{2}\left(x\right)<-f_{1}\left(x\right)+h_{2}\left(x\right)$$

The same can be repeated for ‘m’ iterations until it gets minimized.

#### 3.3.10. Multi-Layer Perceptron Neural Network (MLP-NN)

**Figure 5:**
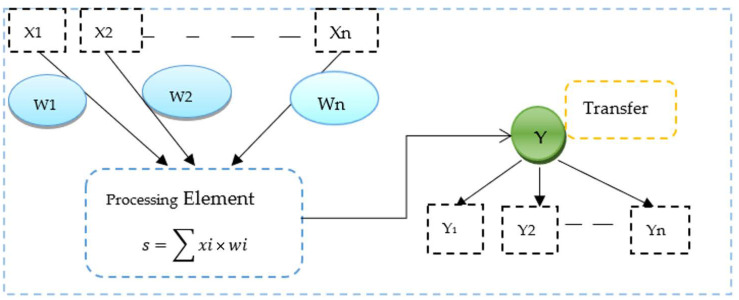
The basic concept of a neural-inspired model.

In this study, we presented a multilayer perceptron neural network (MLP-NN), a feedforward artificial neural network [55] creating outputs from a collection of inputs. A neural network is a computational model inspired by the biological brain. This artificial neuron is called a perceptron as shown in Figure 5, which takes input convolved with weights. X1 and X2 up to Xn are the inputs to the perceptron that are convolved with weights W1 and W2 up to Wn.

**Figure 6:**
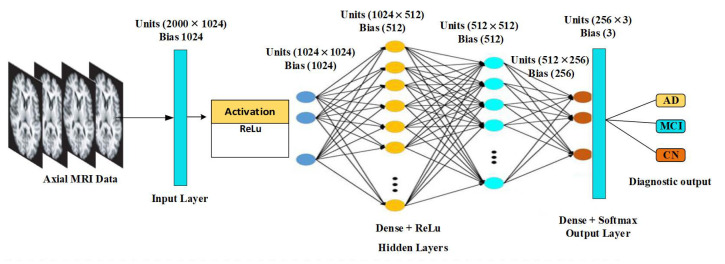
Layers and parameters used in multilayer perceptrons neural network (MLP-NN) architecture.

The activation function takes an input of the calculated weighted sum to pass through it to provide a threshold value. Thus, an artificial neural network involves n such perceptrons. The architectural framework of the proposed MLP-NN is shown in Figure 6. The multi-layer perceptrons (MLPs) with backpropagation learning techniques have input layers with rectified linear units (ReLu), hidden layers with multiple dense units and ReLu activation function and output dense layers with a softmax activation function. The input layer has 2000 × 1024 units with a bias of 1024. Furthermore, four hidden layers (1024 × 1024, 1024 × 512, 512 × 512, and 512 × 256) units are included. Finally, the diagnostic output is generated using the output layer with 253 × 3 units and bias 3.

### 3.4. Proposed Ensemble Learning Approach: XGB + DT + SVM

Based on their performance, the three top-performing learning models, including Extreme Gradient Boosting, Decision Tree and SVM-Poly kernel are chosen for the ensemble learning model in the proposed work. In machine learning, voting ensemble techniques often provide more accurate results than a unified framework due to challenges such as the representation problem, the computing difficulty, and the statistical problem, which can be overcome by employing ensemble approaches. The basic example of stacking is an ensemble learning strategy in which additional innovations are created by combining the projections of many important functional, also known as classifiers, to teach a meta-classifier.

**Figure 7:**
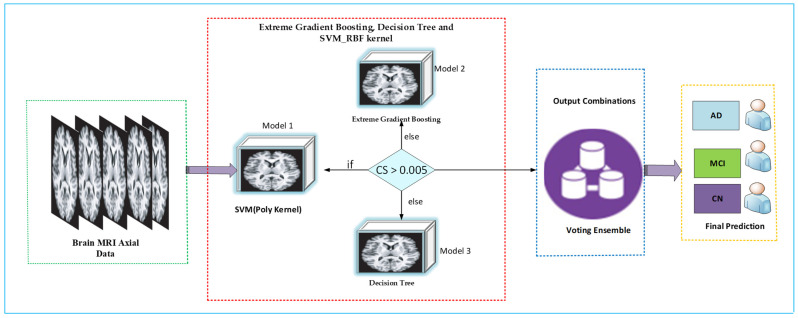
Proposed XGB + DT + SVM Ensemble model.

In this study, we implemented an ensemble of SVM (Poly kernel), Extreme Gradient Boosting Ensemble architecture, and a Decision Tree. This approach increases the performance by combining the advantages of a variety of machine learning architectures combining bagging, boosting and kernel-based rectilinear models such as Decision Tree, Extreme Gradient Boosting and SVM Poly Kernel. XGB + DT + SVM concept incorporates the Variance minimizer from the Decision tree, sampling accuracy enhancer via Extreme Gradient Boosting and Kernel Space Optimization vector using Poly kernel Support Vector Machine to achieve a more accurate average prediction than a single prediction obtained using a single kernel [56]. Any predictor of our selection can serve as the meta-classifier. By splitting the training set into multiple splits for the weak classifiers distribution, it would reduce time complexity or training time, allowing the task to be accomplished easily. Stage one filters are trained with the first split of your training examples. After that, utilize the stage one filters that have been learned to generate predictions about the remaining training splits. After that, the meta-classifier is trained using output from week learners. The confidence score (CS), a regressive value, measures the classification strength using the same feature vectors for classification distinction. The aggregating criteria are a decision of voting for each estimator output that is concatenated. Voting is calculated on the predicted probability of the output class. The architecture of the proposed XGB + DT + SVM is illustrated in Figure 7.

By aggregating the projected class or predicted probability based on voting, this proposed technique significantly improved the model’s performance. Therefore, if many base models are provided to the ensemble voting classifier, it ensures that the mistake is resolved by any model. The challenges of classification include the summation of votes for crisp class labels from different types and the prediction of the most votes in the class. We considered ensemble voting to overcome the error that occurs due to an independent classifier [57,58]. A voting classifier estimator is experimented with to train three kernels (estimators) to predict based on aggregating the findings of each kernel (base estimator).

### 3.5. Grid Search-Based Optimization on ML Methods and Ensemble Technique

The grid Search tuning approach aims to calculate hyperparameter values optimally. In this study, an exhaustive search was conducted on these models’ specific parameter values. This is performed by choosing suitable hyperparameters and tuning them to control the behaviour of the algorithm. The efficiency of our models is optimized by tuning without causing excessive variance or overfitting. Determining the ideal collection of hyperparameters can be computationally challenging. The hyperparameter values were set before learning begins. Regularization parameters in SVMs, k in k-Nearest Neighbors, and hidden layer count in multi-layer perceptron neural networks are other examples. A parameter, on the other hand, constitutes the internal feature of the model and can be measured from the data.

## 4. Experimental Analysis

In this study, a total of 2125 neuroimaging (MRI) data are used, divided into three classes: AD, CN, and MCI. Further, three different forms of classifiers inspired by (machine learning, neural-inspired, and ensemble learning algorithms) are designed for AD classification.

### 4.1. Implementation Setup

We trained our models on the Google Colab framework, providing computational tools and flexibility to adjust utilization limits and hardware availability using Python 3.7, TensorFlow, and the Scikit-learn library. Additionally, we used many Python libraries, including Numpy, Pandas, Matplotlib, and Keras. We also used pydicom, a Python program for working with the DICOM files. TensorFlow and TensorFlow-GPU versions 2.0.0 has also been used.

### 4.2. Performance Evaluation Metrics

The objective of this study is to identify the effectiveness of different algorithms for accurate classification and minimum log loss. Here, different models are investigated to achieve the accuracy, sensitivity, specificity, and log loss values for each classifier measuring the performance of each classifier on the test-set. In the context of statistical classification, a confusion matrix, also known as an error matrix, is used for the visualization of an algorithm’s performance. When evaluating a classification model’s performance on a known set of test data, confusion matrices (also known as contingency tables) are frequently used. The confusion matrix is listed in Table 3.

(a) Accuracy: Classification models can be evaluated based on their accuracy. The fraction of correct predictions made by the model divided by all predictions is referred to as its accuracy as shown in Equation (16).

(16) $$Accuracy=\frac{TP+TN}{TP+TN+FP+FN\;}$$

(b) Sensitivity: The value is represented numerically by dividing the number of correct positive predictions by the total number of positive values, as given in Equation (17).

(17) $$\mathrm{Sensitivity}=\frac{TP\;}{TP+FN}$$

(c) Specificity: The value is mathematically represented by dividing the number of successful negative predictions by the total number of negative values. Sensitivity and specificity are statistical indicators of test result performance. The specificity is computed using the formula given in Equation (18).

(18) $$\mathrm{Specificity}=\frac{TN\;}{TN+FP}$$

(d) Logarithmic loss (log loss): Using a log loss parameter, classification models that yield a probability value between zero and one may be evaluated for their efficiency. Increasing the logarithmic loss value is associated with increasing the anticipated probability value. The log loss action considers the confidence of the prediction in determining how incorrect classifications can be penalized [59]. The mathematical model used for computing logarithmic loss is shown in Equation (19).

(19) $$Log\;Loss\;=-\frac{1}{N}\;\sum_{i-1}[y_{i}\;Log\;\left(p_{i}\right)+(1-y_{i})\;\mathrm{Log}\;(1-\;p_{i})]$$

where the number of instances is represented by *N*, *y_i_* indicates the output of *i*th instance and *p_i_* represents the probability of 1 and (1 − *p_i_*) is the probability of 0. For the total number of cases, log loss accumulates the likelihood of a sample assuming both states 0 and 1.

The next section presents comprehensive results that were obtained after applying different classification approaches.

## 5. Results

The current section Comparison of classic ML models with proposed architecture on the ADNI data. The details of the respective results obtained by the different classifiers are presented below.

### 5.1. Parameter Optimization Results

Grid search determined the ideal model hyperparameters that lead to the most accurate predictions. Table 4 lists the grid parameters and optimized parameters of the classification models implemented in this study.

### 5.2. Classification Results

#### 5.2.1. Before Hyperparameter Tuning

ML models and proposed XGB + DT + SVM were investigated in the context of AD and MCI classification-based neuroimaging analysis. The performance analysis based on accuracy, sensitivity, specificity, and log loss for three classes (AD, CN, and MCI) is given in Table 5. The NB prediction obtained the lowest accuracy of 72.22%, 77.98% and 77.58%, respectively.

The prediction model of Among SVM Polynomial achieved accuracies of 87.84%, 86.21% and 81.48%, respectively. The EGB model and GB obtained accuracies for AD 81.56% and 72.25%, respectively. The classification accuracy of the MLP-NN model for AD, CN and MCI is 89.20%, 81.56% and 84.30%, respectively. XGB + DT + SVM achieved better accuracy at 87.00%, 90.51% and 90.51% for AD, CN and MCI, respectively, compared to other algorithms. Overall, XGB + DT + SVM achieved an average accuracy of 89.77%. The sensitivity values for each class show the test’s ability to correctly identify the class in test data. Among the ML algorithms, SVM Polynomial and the Proposed model have the lowest log loss value of 0.29615 and 0.27795, respectively. Whereas Naive Bayes has the highest log loss value of 13.69922.

#### 5.2.2. After Hyperparameter Tuning

Additionally, Table 6 compares the overall information scores results obtained with optimized parameters for ML classifiers and the proposed ensemble (XGB + DT + SVM) model. GridSearch enhanced the classification performance of these models by choosing the optimized gird of parameters. MLP-NN has shown better classification accuracy of 89.71% among ML models. The proposed ensemble model XGB + DT + SVM with hyperparameter tuning obtained the highest classification average accuracy of 95.75%, which outperformed all the models implemented in this study. XGB + DT + SVM achieved better accuracy 96.12%, 95% and 96.15% for AD, CN and MCI, respectively, compared to other algorithms The conjunction of results after the hyperparameter tuning reduces the variance in the categorical sensitivity and specificity and keeps a reasonable increase for all the different models.

XGB + DT + SVM used traditional, bagging and boosting approaches. The advantage of using traditional techniques is to ensure wide-scale globalization of features. For example, Boosting ensures heard features can be used to decode samples while Bagging ensures there are no overfitting biases. Using an ensemble method, we were able to lower the standard deviation and maintain high precision.

**Figure 8:**
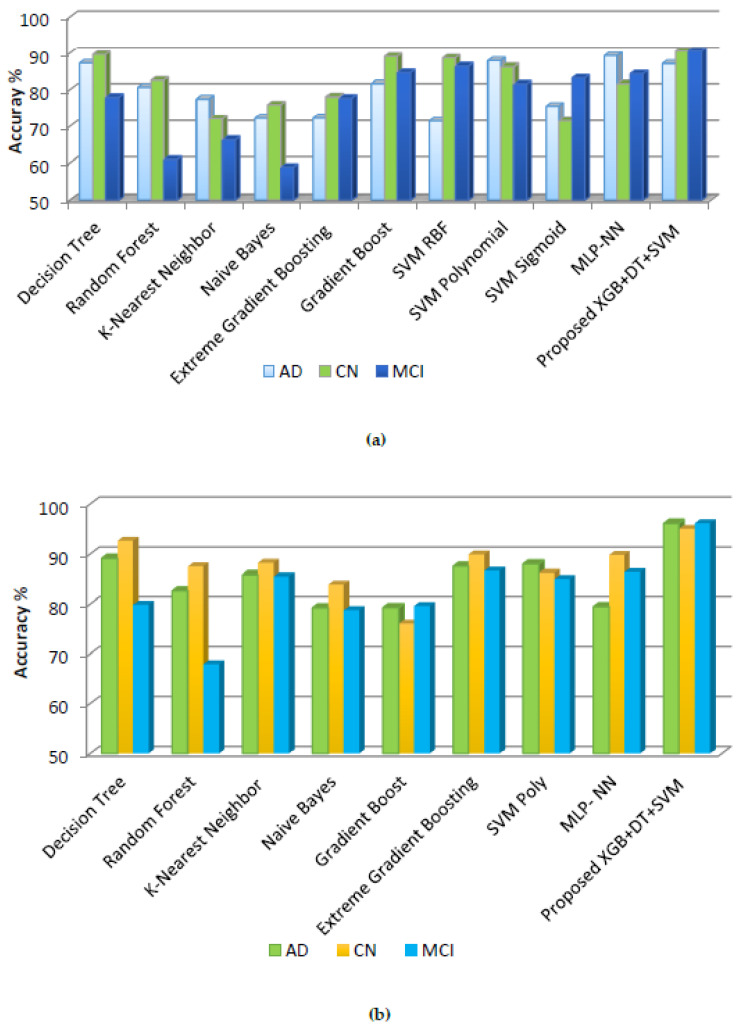
Comparative performance evaluations among classical ML models and XGB + DT + SVM ensemble based on classification accuracy (**a**) before using Grid Search, (**b**) after using Grid Search.

Figure 8 further illustrates the comparative plot, which offers a visual benchmark for contrasting the ML models and proposed model XGB + DT + SVM based on classification accuracy (before and after hyperparameter tuning).

In Figure 8, the authors compared the performance improvement among the models before and after GridSearch. During hyperparameter optimization with GridSearch, the SVM polynomial kernel was chosen among the RBF, polynomial, and sigmoid kernels. Figure 8b represents the performance of models after using GridSearch which includes the SVM polynomial kernel along with other models, whereas, in Figure 8a, all three SVM kernels are included along with other implemented models. Overall, it is demonstrated that model tuning enhances the performance and classification accuracy of all the models.

### 5.3. Statistical Evaluation of Outcomes

In this experiment, k-fold CV, Friedman’s rank test, and *t*-test were used to statistically validate the performance attained by various models for classifying AD, CN, and MCI.

#### 5.3.1. Ten-Fold Cross-Validation

We performed 10 iterations of the k-fold cross-validation (k = 10) method, where each fold was determined to have a mean response value that was within a certain tolerance of the original value.

Cross-validation using a 10-fold set to tune the model on various splits and find the optimal kernel space boundary. The dataset is divided into 10 folds of roughly the same size. Cross-validation is applied to evaluate the performance of each model after hyperparameter tuning.

Table 7 lists the 10-fold cross-validation for each model reported in this study after parameter tuning. The entire data was randomly divided into 10 folds (k = 10). A larger K value results in a less biased model, whereas a smaller k value is comparable to that of the train-test split strategy. Next, k − 1 folds were used to fit the model, and the kth fold was used for validation. This procedure was repeated until each K-fold functioned as a test set. The average of reported scores is then calculated to validate our models. The analysis from this procedure demonstrates the highest mean value is achieved by XGB + DT + SVM (98.84%), whereas Nave Bayes achieved the lowest (67.04%) mean value for 10-fold validation. Finally, the results obtained demonstrate that hyperparameter tuning, when implemented, yields marginally enhanced accuracies in comparison to the values without optimized parameters.

#### 5.3.2. Friedman’s Rank Test

In this work, the significance of the differences between these classifiers is measured using the Friedman rank test [60]. The Friedman test compares methods based on their rankings and is a non-parameter test that is used to represent a comparison of approaches among one another. In our study, we choose a significance threshold of =0.05.

Models are ranked using Friedman’s test, with the best-performing models placed at the top of the list.

The test determines the model’s average rank by taking into account $M_{rj}$, where $M_{rj}$ is the rank of the *i*th models on the *j*th fold of the dataset. Each model, according to the null hypothesis, displays identical behaviour and, hence, has equivalent precision. Friedman’s rank test is distributed by $x_{FM}^{2}$ having the *r* − 1 degree of freedom can be defined in Equation (20) given below: As seen in Equation (20), the distribution of Friedman’s rank test is $x_{FM}^{2}$ with *r* − 1 degrees of freedom.

(20) $$x_{FM}^{2}=\frac{s}{r\left(r+1\right)}[\sum_{j}M_{j}^{2}+\frac{r\;{\left(r+1\right)}^{2}}{8}]$$

If *s* > 10 or/and *r* > 5, the chi-square distribution can be used to estimate the critical value of Friedman’s test statistics; otherwise, the table is referred to.

The *p*-value obtained after Friedman’s statistics test at α = 0.05 demonstrates that the null hypothesis cannot be accepted and substantiates the claim that there is a meaningful distinction between the various models. The estimated average rank and *p*-value of models are shown in Table 8, which demonstrates that the proposed ensemble method XGB + DT + SVM (with highest rank value = 8) and MLP-NN (with second highest rank value = 6.65) has outstanding accuracy performance than other models used in this study.

#### 5.3.3. *T*-Test

Further, the accuracy mean difference is evaluated using a *t*-test on two experimental settings: (i) before hyperparameter tuning, and (ii) after hyperparameter tuning. A *t*-test is a type of inferential statistic used to determine if there is a significant mean difference in performance accuracy [61]. The mean difference of accuracies obtained before and after hyperparameter tuning, i.e., 76.39% and 80.448% demonstrates the enhanced results of (ii) after hyperparameter tuning, with a high statistical difference of -4.058 at *p* < 0.05 as shown in Table 9.

### 5.4. ROC Demonstration of Models Classification Abilities

In this subsection, we have shown the graphical illustration for the models in three categories. Receiver operating characteristic (ROC) curve plots for all classifiers are presented to indicate positive and negative results. The ROC curve is plotted to visualize how the predicted probabilities are compared to the true status [62].

**Figure 9:**
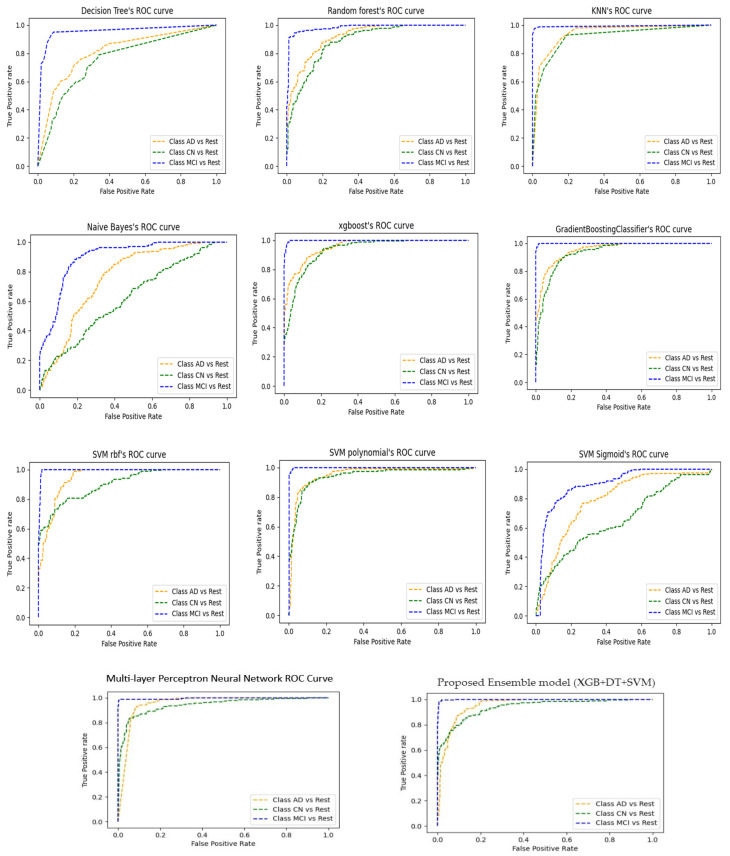
ROC plots for models to visualize the correct predicted and false predicted values for the classification of AD, CN and MCI.

False positive rates are plotted on the X-axis, while true positive rates are plotted on the Y-axis. Anticipated values are shown as TN, TP, FN, and FP on the ROC curve. There are three lines (blue, orange, and green) on the AUC graphs. In each ROC curve, the orange line represents Alzheimer’s disease (AD), the green line represents Control Normal (CN), and the blue line represents Mild Cognitive Impairment (MCI) as shown in Figure 9. The trade-off between sensitivity (TPR) and specificity (1–FPR) is demonstrated in the receiver operating characteristic (ROC) curve. Better results are achieved where the curve is closer to the top-left corner.

XGB + DT + SVM has shown more accurate predictions than other models. MLP-NN showed impressive prediction compared to other ML models. The ROC curve is independent of class distribution. As a result, it is advantageous to test classifiers that predict unusual events such as disease prediction.

### 5.5. Comparison with Existing Studies

In this subsection, the efficacy of the offered models is demonstrated by comparing their performance to that of earlier research. A comprehensive analysis of machine learning algorithms and a proposed approach has been performed for the classification of Alzheimer’s disease.

To ensure the generalization of these models, the efficiency models presented in this study are compared using the accuracy score obtained in previous studies on chosen benchmark dataset [63,64,65,66].

**Figure 10:**
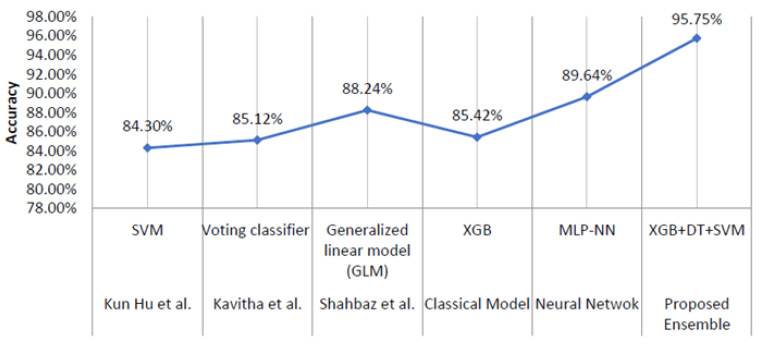
A graphical representation of comparison between best performing XGB proposed XGB + DT + SVM and MLP-NN with previous studies [63,64,65].

Among all the methods analyzed, EGB and MLP-NN have shown better performance. The outcomes of the comparison of EGB and XGB + DT + SVM with previous studies are listed in Table 10. The graphical demonstration of comparison between the results obtained by different models presented in this work with previous studies is shown in Figure 10. Among classical ML models used in this framework, XGB has shown better performance for the classification of AD, CN and MCI with an accuracy of 85.42%. In addition, the proposed XGB + DT + SVM and MLP-NN models outperformed existing approaches with an accuracy of 88.64% and 90.75%, respectively.

## 6. Conclusions

Computer-aided detection (CAD) systems are being developed to help radiologists in interpreting medical imaging findings and minimizing inter-reader variability more precisely. Artificial intelligence and machine learning play an important role in the development of CAD systems as it is extensively used to uncover useful image features from complicated datasets and assist in more reliable diagnosis.

The master model is suggested in this study, and the main principle of this strategy is that when two or more learning procedures combine, the performance of the final model will be improved. Some state-of-the-art learning algorithms and the proposed master model were compared to evaluate the performance of these learning algorithms to classify Alzheimer’s disease from neuroimaging data taken from ADNI. This study tries to improve the understanding of these algorithms and enhance their scope and significance in Alzheimer’s disease classification.

Comparative result analysis showed that after applying GridSearch for parameter optimization, the classification performance improved significantly. Of the various considered algorithms with optimized parameters, MLP-NN outperforms other algorithms with the best classification accuracy of 89.64%. Overall, the proposed XGB + DT + SVM with hyperparameter tuning achieved the highest accuracy of 95.75%, outperforming all the other classifiers on high-resolution brain MRI data.

In this study, statistical analysis of the results was carried out to validate the performance of the proposed models. The analysis has been performed using K-Fold cross-validation, Friedman’s Rank test and *t*-test. These statistical validations ensure the accuracy, effectiveness and efficiency of the models.

Further, the results of the proposed model (XGB + DT + SVM) and other state-of-the-art models considered in this study such as the neural-inspired model (MLP-NN) and XGB were compared with the results of existing studies. The results show that the models proposed in this study have outperformed the results of the existing studies.

## 7. Future Scope

From the state-of-the-art approaches, we can conclude that certain gaps need to be overcome in further research and more investigations are required to perform future work in the direction. There are several challenges identified. Firstly, the experimentation can be performed using a large number of samples to yield improved performance. Secondly, the work can be extended by using different neuroimaging modalities to determine the effective one. Thirdly, deep learning algorithms such as 3D-Convolution Neural Networks (3D-CNN), Recurrent Neural Networks (RNN) and pre-trained transfer learning models such as VGG-16, VGG-19, ResNet can be utilized to address the existing prediction and classification problems for better diagnosis. Fourthly, the AD data can be experimented with by including more viewing planes such as sagittal and coronal instead of considering just one single plane. Fifthly, the use of nature-inspired algorithms can be experimented with for optimization and also extract the relevant features from the data. Moreover, the work can be extended by considering the severity levels of AD, i.e., early mild cognitive impairment (EMCI), late mild cognitive impairment (LMCI), static mild cognitive impairment (SMCI) and progressive mild cognitive impairment (PMCI).

## Figures and Tables

**Table 1:** A literature review. The acronym used is provided in the Appendix A of the paper.

Author	Data Base	Imaging Modality	Total Subjects	Dataset Division	Feature Extraction and Classification Techniques	Classification Accuracy
[25]	ADNI	s-MRI	259	sMCI = 139; pMCI = 120	FW LLE	sMCI/pMCI = 80.0%
[29]	ADNI	MRI	710	AD = 200; MCI = 280; pMCI = 120; sMCI = 160; CN = 230	MK-Boost	AD/CN = 94.65% MCI/CN = 85.79% sMCI/pMCI = 72.08%
[31]	ADNI	MRI, PET	726	AD = 101; CI = 223; sMCI = 93; pMCI = 140; CN= 169	SVM	AD/CN = 91.20% MCI/CN = 76.40% sMCI/pMCI = 74.20%
[32]	ADNI	s-MRI	303	AD = 158; CN = 145	SVM-RFE SVM	AD/CN = 90.76%
[35]	ADNI	MRI, PET	654	AD = 154; MCI = 346; CN = 154	RFLD SVM	AD/CN = 88.30% MCI/CN = 79.02%
[36]	ADNI	s-MRI, PET	340	AD = 113; MCI = 110; CN = 117	AAL, ROI mSCDDL	AD/CN = 98.5% MCI/CN = 82.8%
[37]	ADNI	MRI, PET	427	AD = 65; pMCI = 95; sMCI = 132; CN = 135	MDS, PCA SVM	AD/CN = 96.5% MCI/CN = 91.74% sMCI/pMCI =88.99%
[38]	ADNI	MRI, PET	249	AD = 70; MCI = 111; CN = 68	PCA SVM	AD/CN = 92% MCI/CN = 84%
[39]	OASIS, LH	MRI	196	AD = 98; CN = 98	3AF 8L-CNN	AD/CN = 97.65%
[40]	ADNI	MRI	710	AD = 200; pMCI = 120; sMCI = 160; CN = 230	ROI, FS MK-BOOST	AD/CN = 95.24% MCI/CN = 86.35% sMCI/pMCI = 74.25%
[41]	ADNI1	MRI	891	AD = 199; pMCI = 167; sMCI = 226; CN = 229	ROI, VBM, CLM LDMIL	AD/CN = 95.86% sMCI/pMCI = 77.64%
	ADNI2	MRI	636	AD = 159; pMCI = 38; sMCI = 239; CN = 200		
	MIRIAD	MRI	69	AD = 23; CN = 46		AD/CN = 97.16%
[42]	SELF	rs-MRI	68	AD = 34; CN = 34	3LHPM-ICA DL Algo.	AD/CN = 95.59%
[28]	ADNI	MRI FDG- PET	1628	AD = 342; MCI = 418; CN = 866	RVB, SUVR, ROI L-SVM, LR, RF	AD/CN = 88% MCI/CN = 80% sMCI/pMCI = 73%
	AIBL		139	AD = 72; MCI = 94; pMCI = 21; sMCI = 16; CN = 442		
	OASIS		416	AD = 100; CN = 93		

**Table 2:** Detailed Sample-wise Distribution of Dataset.

Dataset	Total	Alzheimer’s Disease	Mild Cognitive Impairment	Control Normal
Brain Axial MRI T1 and T2 weighted	2127	Images = 612	Images = 538	Images = 975
		Class 1 (AD)	Class 2 (MCI)	Class 3 (CN)
		Label = 0	Label = 1	Label = 2

**Table 3:** Overview of confusion matrix/contingency table.

	Ground Truth/Actual		
**Predicted**		**Actual Positive**	**Actual Negative**
	**Predicted Positive**	True positive (TP)	False Negative (FN)
	**Predicted Negative**	False Positive (FP)	True Negative (TN)

**Table 4:** Hyperparameter tuning to obtain optimized parameters using GridSearch.

Model	Grid Parameters	Optimized Parameters
Decision Tree	parameters = { ‘max_depth’: [2,4,6,8,10,12,14], ‘min_samples_leaf’: [5,7,9,11,13,15], ‘criterion’: [“gini”, “entropy”], ‘min_samples_split’: [1,2,3,4,5,6,7,8]}	parameters = { ‘max_depth’: [10], ‘min_samples_leaf’: [15], ‘criterion’: [‘gini’], ‘min_samples_split’: [4]}
Random Forest	model_params = { ‘n_estimators’: randint(4200), ‘max_features’: truncnorm(a = 0, B = 1, loc = 0.25, scale = 0.1), ‘min_samples_split’:uniform (0.01,0.199)}	model_params = { ‘n_estimators’: [148], ‘max_features’: [0.276163], ‘min_samples_split’: [0.0392]}
K-Nearest Neighbor	grid_params = { ‘n_neighbors’: [3,5,11,13,15], ‘weights’: [‘uniform’,’distance’], ‘metric’: [‘minkowski’,’euclidean’,’manhattan’]}	grid_params = { ‘n_neighbors’: [3], ‘weights’: [‘uniform’], ‘metric’: [‘minkowski’]}
Naïve Bayes	parameters = { ‘Var_smoothing’:np.logspace (0,9,num = 1000)}	parameters = { ‘Var_smoothing’: [4.328]}
Extreme Gradient Boosting	parameters = { ‘learning_rate’: [0.01,0.1], ‘max_depth’: [3,5,6,10], ‘subsample’: [0.5,0.7], ‘colsample_bytree’: [0.5,0.7], ‘n_estimators’: [100,200,500], ‘objective’: [‘reg:squarederror’]}	parameters = { ‘learning_rate’: [0.1], ‘max_depth’: [6], ‘subsample’: [0.5], ‘colsample_bytree’: [0.5], ‘n_estimators’: [100], ‘objective’: [‘reg:squarederror’]}
Gradient Boost	parameters = { “n_estimators”: [50,150], “max_depth”: [1,3,5], “learning_rate”: [0.01,0.1,0.5,1], ‘max_depth’: [3,5,6,10], ‘min_child_weight’: [1,3,5], ‘subsample’: [0.5,0.7], ‘colsample_bytree’: [0.5,0.7], ‘n_estimators’: [100,200,500],}	parameters = { “n_estimators”: [80], “max_depth”: [3], “learning_rate”: [0.5], ‘max_depth’: [6], ‘min_child_weight’: [5], ‘subsample’: [0.5], ‘colsample_bytree’: [0.5], ‘n_estimators’: [100]}
Multi-layer Perceptron Neural Network (MLP-NN)	Parameters = {‘batch_size’: [20,30], ‘nb_epoch’: [50,100,150], ‘optimizer’: [‘adam’,’rmsprop’,’SGD’] ‘units’: [512,1024]}	parameters = {‘batch_size’: [20], ‘nb_epoch’: [50], ‘optimizer’: [‘adam’], ‘units’: [512]}
Proposed Ensemble model XGB + DT + SVM	parameters = { ‘learning_rate’: [0.01,0.1], ‘max_depth’: [3,5,6,10], ‘min_child_weight’: [1,3,5], ‘subsample’: [0.5,0.7], ‘colsample_bytree’: [0.5,0.7], ‘n_estimators’: [100,200,500], ‘objective’: [‘reg:squarederror’], ‘min_samples_leaf’: [5,7,9,11,13,15], ‘criterion’: [“gini”, “entropy”], ‘min_samples_split’: [1,2,3,4,5,6,7,8] ‘kernel’: [‘rbf’, ‘poly’, ‘sigmoid’]}	param_grid = { “n_estimators”: [80], “max_depth”: [3], “learning_rate”: [0.5], ‘max_depth’: [6], ‘min_child_weight’: [5], ‘subsample’: [0.5], ‘colsample_bytree’: [0.5], ‘n_estimators’: [100] ‘kernel’: [‘rbf’]}

**Table 5:** Before hyperparameter tuning (parameter optimization) using GridSearch.

Model	Accuracy (%)			Sensitivity (%)			Specificity (%)			Log Loss
	AD	CN	MCI	AD	CN	MCI	AD	CN	MCI	
DT	87.12	89.56	77.84	95.22	92.39	87.57	49.06	98.82	90.24	0.74054
RF	80.59	82.51	60.89	75.76	53.26	79.50	71.29	81.58	94.11	0.71971
K-NN	77.42	72.09	66.30	83.61	46.63	80.12	61.16	96.39	76.96	0.38076
NB	72.22	75.86	58.75	72.01	92.39	91.30	57.74	95.46	64.40	13.6992
GB	72.25	77.98	77.58	89.76	48.91	88.19	72.27	90.00	97.51	0.53312
XGB	81.56	89.00	84.63	92.49	92.49	97.51	80.05	94.27	98.71	0.36665
SVM RBF	71.45	88.59	86.36	98.63	54.89	99.00	79.63	99.11	96.05	0.33290
SVM Poly	87.84	86.21	81.48	83.95	58.15	94.40	79.20	89.23	95.40	0.29615
SVM Sigmoid	75.48	71.49	83.14	90.44	76.08	96.27	86.51	93.54	99.26	0.48208
MLP-NN	89.20	81.56	84.30	95.22	64.67	98.75	82.49	96.47	98.51	0.36665
Proposed XGB + DT + SVM	87.00	90.51	90.51	91.80	77.17	98.75	88.26	94.27	99.51	0.27795

**Table 6:** After hyperparameter tuning (parameter optimization) using GridSearch.

Model	Accuracy (%)			Sensitivity (%)			Specificity (%)			Log Loss
	AD	CN	MCI	AD	CN	MCI	AD	CN	MCI	
DT	89.12	92.56	79.84	97.26	37.50	91.92	64.77	97.30	98.33	0.58883
RF	82.59	87.51	67.89	76.79	57.06	81.36	74.68	83.17	92.96	0.71971
K-NN	85.84	88.21	85.48	90.44	69.02	97.51	83.77	93.56	98.74	1.31750
NB	79.22	83.86	78.75	85.32a	75.43	89.44	50.00	99.87	71.42	0.27213
GB	79.25	75.98	79.58	91.46	55.43	93.78	76.43	93.11	97.88	0.39457
XGB	87.56	89.89	86.63	93.85	62.50	96.27	79.88	79.88	99.48	0.32501
SVM Poly	88.00	86.21	85.00	92.75	77.54	87.00	86.00	81.76	84.34	0.31123
MLP- NN	79.45	89.79	86.36	90.78	77.17	96.27	87.09	93.76	99.27	0.42902
Proposed XGB + DT + SVM	96.12	95	96.15	93.85	78.80	98.75	89.67	95.59	99.05	0.24926

**Table 7:** Ten-fold cross-validation after parameter optimization (hyperparameter tuning).

Folds	DT	RF	K-NN	NB	SVM-RBF	SVM- Poly	SVM- Sigmoid	GB	XGB	MLP-NN	Proposed XGB + DT + SVM
**1_fold**	0.71812	0.77181	0.83892	0.83892	0.83221	0.83221	0.65771	0.81879	0.83892	0.84563	0.98547
**2_fold**	0.71812	0.79865	0.90604	0.89261	0.92617	0.92617	0.67782	0.79867	0.87248	0.93288	0.97564
**3_fold**	0.67114	0.73825	0.86577	0.80536	0.87919	0.87919	0.70469	0.83892	0.82550	0.87248	0.97554
**4_fold**	0.67785	0.83221	0.91275	0.89932	0.87919	0.87919	0.77852	0.86577	0.87919	0.87919	0.96587
**5_fold**	0.71812	0.79865	0.87919	0.89261	0.87248	0.87248	0.63087	0.81208	0.83892	0.88590	0.98658
**6_fold**	0.71812	0.75838	0.85234	0.82550	0.88590	0.88590	0.65771	0.80536	0.89261	0.89261	0.97857
**7_fold**	0.67785	0.79194	0.89932	0.87248	0.90604	0.90604	0.66442	0.84563	0.87919	0.91946	0.98143
**8_fold**	0.67567	0.79054	0.88513	0.82432	0.87837	0.87837	0.68243	0.82432	0.87837	0.87837	0.98543
**9_fold**	0.68243	0.79054	0.84459	0.81756	0.89189	0.89189	0.62162	0.84459	0.84459	0.89189	0.99948
**10_fold**	0.75675	0.76845	0.86486	0.85810	0.87837	0.87837	0.64864	0.80405	0.86486	0.88513	0.96854
**Mean**	**0.70141**	**0.78394**	**0.87489**	**0.85268**	**0.87424**	**0.87424**	**0.67043**	**0.82582**	**0.86146**	**0.88835**	**0.98845**

**Table 8:** Utilizing Friedman’s Rank test to determine each model’s mean rank position and *p*-value.

Rank	1st	2nd	3rd	4th	5th	6th	7th	8th	*p*-Value
Model	GB	RF	K-NN	NB	DT	XGB	MLP-NN	XGB + DT + SVM	4.36 × 10^−12^
Average rank	2	3.05	5.25	1	4.15	5.9	6.65	8	

**Table 9:** Mean differences of accuracy before and after hyperparameter tuning using *t*-test.

	Before Hyperparameter Tuning	After Hyperparameter Tuning	Mean Difference	*t*-Values
Accuracy (%)	76.39	80.448	−4.058 **	−3.225

Note: (**) indicate the significance level at a 5% level of significance.

**Table 10:** Comparative analysis of presented models with previous studies.

S. No	Study Reference	Dataset	Technique	Accuracy
1.	Kun Hu et al. [67]	ADNI	SVM	84.30%
2.	Kavitha et al. [68]	OASIS	Voting classifier	85.12%
3.	Shahbaz, Muhammad et al. [69]	ADNI	Generalized linear model (GLM)	88.24%
4.	Classical ML Model	ADNI	XGB	85.42%
5.	Neural Network	ADNI	MLP-NN	89.64%
6.	Proposed Voting Ensemble	ADNI	XGB + DT + SVM	95.75%

## Data Availability

The dataset available online can be downloaded from the links given as follows. ADNI DataSet at the URL: https://ida.loni.usc.edu/login.jsp?project=ADNI (accessed on 10 July 2022).

## Appendix A

The acronyms for labels used in Table 1 are enlisted in Table A1.

**Table A1:** Acronym for labels used in Table 1.

Labels	Acronym	Labels	Acronym
**AF**	Activation functions	**8L-CNN**	8-layer convolution neural network
**AIBL**	Australian Imaging, Biomarker & Lifestyle Flagship Study of Ageing	**MIRIAD**	Minimal Interval Resonance Imaging in Alzheimer’s Disease dataset
**LDMIL**	landmark-based deep multi-instance learning	**VBM**	Voxel-based morphometry
**CLM**	Conventional landmark-based morphometry	**MMSR**	Multi-modal sparse representation
**SPA**	Sparse patch-based approach	**DL Algo**	Deep learning algorithm
**FS**	Fisher score	**RVB**	Regional Voxel-based method
**SUVR**	Standardized uptake value ratio	**GGML**	Graph-guided multitask-task learning
**N-CK**	N-number (combination of multi-kernels)	**E-MK**	Ensemble multi-kernel
**RFE**	Recursive feature elimination	**OASIS**	Open Access Series of Imaging Studies
**RFES**	Random forest-based ensemble strategy	**GIM**	Gini importance measure
**lMRI**	longitudinal-MRI	**RFLD**	Regression forest-based landmark detection model
**AAL**	Automated Anatomical Labeling	**FW**	Filter Wrapper method
**N-CK**	N-number (combination of multi-kernels)	**DMTFS**	Discriminative Multi-Task Feature Selection
**FA**	Fisher Algorithm	**WA**	Wrapper Algorithm
**MK-SVM**	Multi kernel- SVM	**PM-Kernel**	Probabilistic multi-kernel
**p-MCI**	Progressive-MCI	**L-SVM**	Lagrangian-SVM
**s-MCI**	Static-MCI	**LR, RF**	Logistic regression, Random Forest
